# Comparative Evaluation of Polyphenol Contents and Antioxidant Activities between Ethanol Extracts of *Vitex negundo* and *Vitex trifolia* L. Leaves by Different Methods

**DOI:** 10.3390/plants6040045

**Published:** 2017-09-27

**Authors:** Sarla Saklani, Abhay Prakash Mishra, Harish Chandra, Maria Stefanova Atanassova, Milan Stankovic, Bhawana Sati, Mohammad Ali Shariati, Manisha Nigam, Mohammad Usman Khan, Sergey Plygun, Hicham Elmsellem, Hafiz Ansar Rasul Suleria

**Affiliations:** 1 Department of Pharmaceutical Chemistry, H. N. B. Garhwal (A Central) University, Srinagar Garhwal, Uttarakhand 246174, India; pharmachemhnbgu@gmail.com; 2 High Altitude Plant Physiology Research Centre, H. N. B. Garhwal (A Central) University, Srinagar Garhwal, Uttarakhand 246174, India; 3 Scientific Consulting, Chemical Engineering, University of Chemical Technology and Metallurgy (UCTM), Sofia 1734, Bulgaria; msatanassova@abv.bg; 4 Department of Biology and Ecology, Faculty of Science, University of Kragujevac, Radoja Domanovića No. 12, Kragujevac 34000, Serbia; mstankovic@kg.ac.rs; 5 Department of Pharmacy, Banasthali Vidyapeeth, Rajasthan 304022, India; bhawana.sati@gmail.com; 6 Department of Scientific affairs, Kurks State Agricultural Academy, Kurks 305021, Russia; shariatymohammadali@gmail.com; 7 All-Russian Research Institute of Phytopathology, Moscow, Bolshie Vyazemy 143050, Russia; rjoas@yandex.ru; 8 Department of Biochemistry, H. N. B. Garhwal (A Central) University, Srinagar Garhwal, Uttarakhand 246174, India; anandmanisha23@gmail.com; 9 Department of Biological Systems Engineering, Bio Product Sciences and Engineering Laboratory (BSEL), Washington State University, 2710 Crimson Way, Richland, WA 99354-1671, USA; engineer_usman_khan@yahoo.com; 10 Laboratoire de Chimie Analytique Appliquée, Matériaux et Environnement (LC2AME), Faculté des Sciences, B.P. 717, Oujda 60000, Morocco; h.elmsellem@gmail.com; 11 UQ Diamantina Institute, Translational Research Institute, Faculty of Medicine, The University of Queensland, 37 Kent Street Woolloongabba, Brisbane, QLD 4102, Australia; hafiz.suleria@uqconnect.edu.au; 12 Department of Food, Nutrition, Dietetics & Health, Kansas State University, Manhattan, KS 66506, USA

**Keywords:** phytochemicals, Nirgundi, chaste tree, antibacterial, free radical, scavenger, oxidative stress

## Abstract

The in vitro antioxidant potential assay between ethanolic extracts of two species from the genus *Vitex* (*Vitex negundo* L. and *Vitex trifolia* L.) belonging to the Lamiaceae family were evaluated. The antioxidant properties of different extracts prepared from both plant species were evaluated by different methods. DPPH scavenging, nitric oxide scavenging, and β-carotene-linoleic acid and ferrous ion chelation methods were applied. The antioxidant activities of these two species were compared to standard antioxidants such as butylated hydroxytoluene (BHT), ascorbic acid, and Ethylene diamine tetra acetic acid (EDTA). Both species of *Vitex* showed significant antioxidant activity in all of the tested methods. As compared to *V. trifolia* L. (60.87–89.99%; 40.0–226.7 μg/mL), *V. negundo* has been found to hold higher antioxidant activity (62.6–94.22%; IC_50_ = 23.5–208.3 μg/mL) in all assays. In accordance with antioxidant activity, total polyphenol contents in *V. negundo* possessed greater phenolic (89.71 mg GAE/g dry weight of extract) and flavonoid content (63.11 mg QE/g dry weight of extract) as compared to that of *V. trifolia* (77.20 mg GAE/g and 57.41 mg QE/g dry weight of extract respectively). Our study revealed the significant correlation between the antioxidant activity and total phenolic and flavonoid contents of both plant species.

## 1. Introduction

The genus *Vitex* (Lamiaceae) contains 270 species with diverse medicinal active constituents and properties. These species are predominantly trees and shrubs, found in tropical and subtropical regions. Other species hove fruits, seeds, and roots that are also important in traditional medicines. Some of *Vitex* species, including *Vitex negundo*, *Vitex glabrata*, *Vitex leucoxylon*, *Vitex penduncularis*, *Vitex pinnata*, and *Vitex trifolia*, are found in India [1]. These species are commonly used in traditional medicine to treat a wide range of ailments, such as depression, venereal diseases, asthma, allergy, skin diseases, snakebite, and body pains [2,3]. Many plants of the genus *Vitex* are used for their interesting biological activities, such as treatment of cough, wound healing, larvicidal, anti-HIV, anticancer, and trypanocidal [4,5,6].

*V. negundo* L. (*Lamiaceae*), known as Chinese chastetree, Sambhalu, or Nirgundi (in Ayurveda), grows gregariously in wastelands and is cultivated as a hedge-plant. The leaf extract of *V. negundo* is generally used as a grain preservation material to protect pulses against insects [7]. It contains many polyphenolic compounds, terpenoids, glycosidic iridoids, and alkaloids. Among its chemical constituents, it has several flavonoids such as casticin, orientin, isoorientin, luteolin, lutein-7-*O*-glucoside, corymbosin, gardenins A and B, 3-Odesmethylartemetin, 5-Odesmethylnobiletin, and 3′,4′,5,5′,6,7,8-heptamethyoxyflavone. Interestingly, it is used conventionally for the treatment of eye-disease, toothache, inflammation, leukoderma, enlargement of the spleen, skin-ulcers, in catarrhal fever, rheumatoid arthritis, gonorrhea, and bronchitis. Moreover, it is also used as a tonic, vermifuge, lactagogue, emmenagogue, antioxidant, antibacterial, antipyretic, and antihistaminic agent. The oil of *V. negundo* has beneficial effect when applied to sinuses and scrofulous sores. Its extract has been reported to possess antitumor activity against Dalton ascites tumor cells in Swiss albino mice [8]. Lagundi tablets prepared from leaves of *V. negundo*, and commercially marketed as Ascof^®^ (Rose Pharmacy, Mandaue, Philippines) are prescribed for the relief of mild to moderate bronchial asthma and cough [9].

*V. trifolia* L. (*Lamiaceae*) is commonly known as a chaste tree. It is a deciduous shrub found in tropical and subtropical regions. The plant species is native to Southeast Asia, Micronesia, Australia, and East Africa. This plant can be commonly found along the banks of water bodies like canals, rivers, and ponds. It is known to produce a variety of diterpenoids that display antioxidant, cytotoxic, and trypanosidal activities [10]. *V. trifolia* is conventionally consumed to improve memory, relieve pain, remove the bad taste in mouth, cure fever, and as a diabetes, amenorrhea, and cancer treatment. The flowers of *V. trifolia* mixed with honey are used to treat fever accompanied by vomiting and severe thirst. Additionally, it is used as an antibacterial, a sedative, and to treat rheumatism and the common cold in Asian countries [11,12,13]. 

Although all parts of *Vitex* species are used as medicament in different indigenous systems of medicine, the leaves are most potent for medicinal use. Hence, the basic aim of the present study was executed to explore the comparative account of the total polyphenolic contents and as well as the antioxidant activity for ethanolic extracts of *V. negundo* and *V. trifolia* (leaves) using a plethora of antioxidant assays.

## 2. Results

Phytochemical screening of the leaf extracts of *V. negundo* and *V. trifolia* revealed the presence of different phytochemicals, as summarized in Table 1. For both plants, a range of extracting solvents (petroleum ether, chloroform, ethyl acetate, ethanol, and water) were used. Out of tested solvents, ethanol was proven to be excellent for the extraction of phytochemicals as evident from the results (Table 1). Alkaloids were not detected in the petroleum ether extract of *V. negundo*. Water extract showed the presence of carbohydrates and tannins in both plants. Saponin was detected in ethanol, water, and petroleum ether extracts of *V. negundo*. However, in the case of *V. trifolia,* saponin was only detected in ethanol extracts. *V. trifolia* leave extract had proven to be a good source of flavonoids. The qualitative chemical screening test confirmed that the ethanol extract showed maximum phytoconstituents including flavonoids mostly responsible for antioxidant activity in the *V. negundo*.

### 2.1. Total Phenolic and Total Flavonoid Contents (TPC and TFC)

TPC in the ethanol extract of *V. negundo* and *V. trifolia* leaves extracts using the Folin-Ciocalteu reagent is expressed in terms of gallic acid equivalent or GAE (the standard curve equation: y = 6.019x − 0.0186, r^2^ = 0.989) as mg GAE/g of extract. The concentrations of flavonoids are expressed in terms of quercetin equivalent (QE) (the standard curve equation: y = 15.121x − 0.0472, r^2^ = 0.986), as mg QE/g of extract (Table 2). The ethanol extract of *V. negundo* leaves exhibited the higher content of total phenolics (89.71 mg GAE/g) and total flavonoids (63.11 mg QE/g) as compared to the ethanolic extract of *V. trifolia*, which have TPC (77.20 mg GAE/g) and TFC (57.41 mg QE/g).

### 2.2. Antioxidant Activity

The antioxidant activity of ethanol extracts from both plant species is expressed in terms of percentage of inhibition (%) and IC_50_ values (μg/mL).

#### 2.2.1. DPPH Free Radical-Scavenging Assay

**Figure 1:**
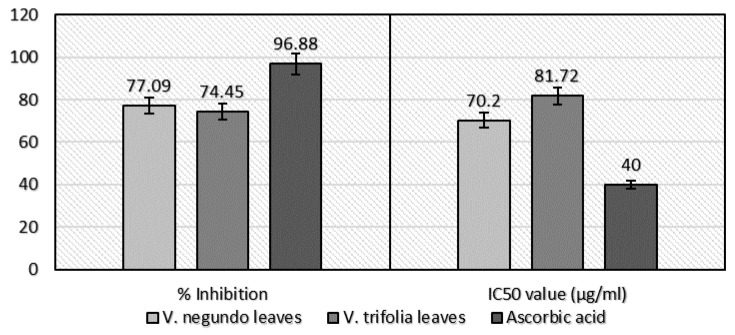
2,2-Diphenyl-1-picrylhydrazyl (DPPH) assay of *V. negundo* and *V. trifolia* (leaves) ethanol extract.

To evaluate the scavenging effect of DPPH·+ in ethanol extract of *V. negundo* and *V. trifolia* leaves, DPPH·+ inhibition was investigated, and these results were shown as relative activities against control. The extracts constituted from the leaves of *V. negundo* and *V. trifolia* showed different antioxidant potential. Crude ethanol extract of the leaves of *V. negundo* and *V. trifolia* leaves and ascorbic acid (IC_50_ = 40.00 μg/mL) showed to have a potent antioxidant activity. A lower IC_50_ value indicates higher antioxidant potential. Both extracts have been shown to have significant DPPH radical scavenging activity (Figure 1). The *V. negundo* leaf ethanol extract was found to be the richest source of antioxidants among the samples investigated. The IC_50_ value of the *V. negundo* leaf ethanol extract was found to be 77.09% (IC_50_ = 70.20 μg/mL), which is lower than that of the *V. trifolia* leaf, which has a scavenging activity of 74.45% (IC_50_ = 81.72 μg/mL). In addition, we compared the antioxidant potential of our samples with that of vitamin C (ascorbic acid). The same procedure was applied to vitamin C, and its IC_50_ value was determined. Despite the scavenging activity of ascorbic acid (96.88%), a well-known antioxidant was fairly more prominent than that of extracts. 

#### 2.2.2. β-Carotene-Linoleic Acid Assay 

**Figure 2:**
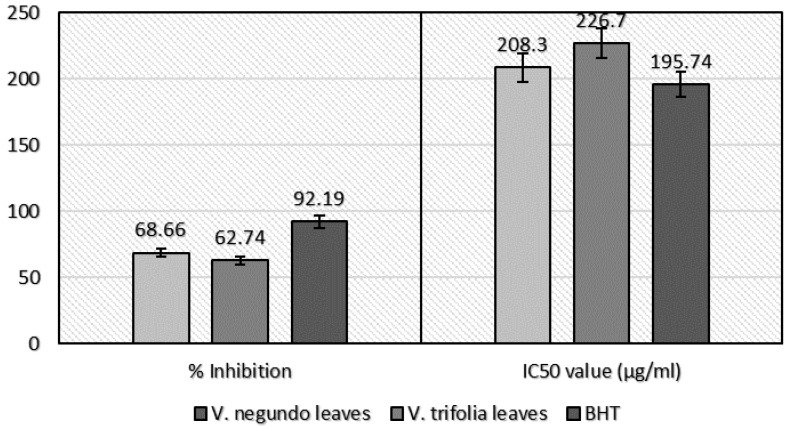
β-Carotene assay of *V. negundo* and *V. trifolia* (leaves) ethanol extract.

The inhibition extent of lipid oxidation by extracts (*V. negundo* and *V. trifolia* leaves) when compared to BHT showed significant activity (Figure 2). The higher antioxidant activity was observed in *V. negundo* leaves (68.66%) as compared to *V. trifolia* leaves (62.74%), with an IC_50_ value of 208.3 μg/mL and 226.7 μg/mL, respectively. The antioxidant capacity of standard BHT was 92.19% at 195.74 μg/mL IC_50_ value.

#### 2.2.3. Nitric Oxide Radical Scavenging Assay

**Figure 3:**
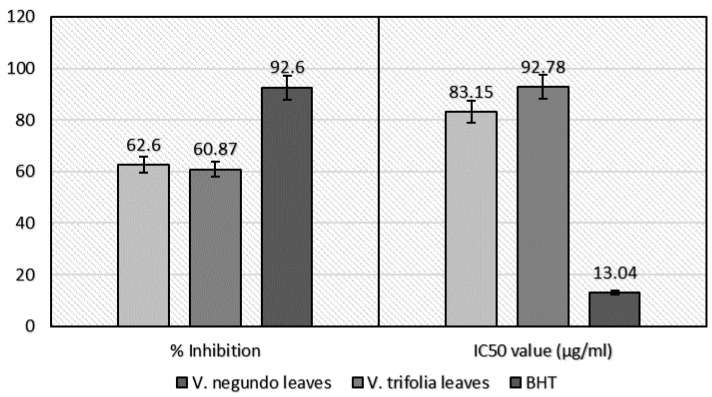
Nitric oxide (NO) scavenging assay of *V. negundo* and *V. trifolia* (leaves) ethanol extract.

The current study proved that the extracts studied had comparable nitric oxide scavenging activity with the standard BHT (Figure 3). It was observed that the scavenging percentage of nitric oxide in the *V. negundo* leaves was 62.60% with an IC_50_ value of 83.15 μg/mL, whereas in *V. trifolia* leaves was 60.87% over 92.78 μg/mL IC_50_ value. An amount of 13.04 μg/mL BHT was needed to obtain 50% inhibition. The IC_50_ value of the composed extracts was greater than that of the standard, which showed lower activity of extracts than the standard. Interestingly, in this assay, both plant extracts exhibited nitric oxide scavenging activity, which was moderately similar to each in terms of percentage.

#### 2.2.4. Ferrous Ion Chelating Activity

**Figure 4:**
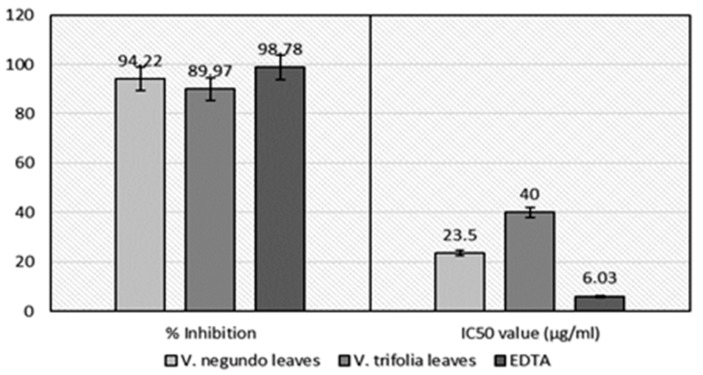
Ferrous ion chelating assay of *V. negundo* and *V. trifolia* (leaves) ethanol extract.

EDTA is a well-known metal ion chelator, therefore, the chelating effect of *V. negundo* and *V. trifolia* leaves extracts was compared with it. Both extracts interfered with the formation of ferrous and ferrozine complex, suggesting that they had chelating activity. The strongest iron chelating activity of the extracts was noticed as 94.22% (IC_50_ = 23.5 μg/mL) in *V. negundo* and 89.97% (IC_50_ = 40.0 μg/mL) in *V. trifolia,* when compared with EDTA (98.78%, IC_50_ = 6.03 μg/mL), as shown in Figure 4. 

## 3. Discussion

*Vitex* species, an abundant herb/tree in the Indian subcontinent, possess great medicinal value. Therefore, it can be exploited for many herbal drugs therapeutics. The present study was carried out to compare the antioxidant potential of both species i.e., *V. negundo* and *V. trifolia*. Our study suggests that both plants have significant antioxidant potential, and both species can be exploited equally for preparation of Ayurvedic drugs or herbal drugs. Antioxidant properties imparting in any herbal preparation can be prescribed for premature skin aging for skin cancer, improving the immune system, removing free radicals from the body, eye health, troubles of memory, and so forth. 

DPPH radical scavenging, β-carotene-linoleic acid assay, nitric oxide radical scavenging assay, Ferrous ion chelating activity, determination of total phenolic compounds, and determination of total flavonoid content of the ethanol extracts of the *V. negundo* and *V. trifolia* were examined in this study.

Significant variations were found in total polyphenolic contents of both *Vitex* species. The favorable properties resulting from the presence of TPC in the target species have been ascribed to their antioxidant activity. TPC may contribute directly to the antioxidative action mainly due to their redox properties, which can play an important role in absorbing and neutralizing free radicals, quenching singlet and triplet oxygen, or decomposing peroxides. Flavonoids are the most important natural phenolics and have a large number of biological and chemical properties, including radical scavenging. It has been suggested that up to 1.0 g polyphenolic compounds (from a diet rich in fruits and vegetables) ingested daily have inhibitory effects on mutagenesis and carcinogenesis in humans [14,15]. The presence of flavonoid, phenol, terpenoids, anthraquinones, carbohydrates, and steroids were also previously reported in *Vitex negundo* [16]. Total phenolic content of *V. negundo* was estimated to be 261 mg gram equivalents of catechol of *Vitex negundo*, and the total flavonoid content was expressed in Quercetin gram equivalents of 278 mg equivalents per gram of the extract of *V. negundo* [17]. The presence of phenolic compound in both species contributes to its antioxidant properties. The mechanism of phenolic content for imparting antioxidant properties was due to its neutralizing lipid free radicals and preventing decomposition of hydroperoxides into free radicals [18].

The results from the antioxidant analyses showed that both tested extracts might reach some confident level act as radical scavengers. The antioxidant activity of *V. negundo* and *V. trifolia* leaves extracts were determined using ethanol DPPH solution. This is a widely accepted technique for estimating free radical-scavenging activities of antioxidants. DPPH is a stable nitrogen-centered free radical, the color of which changes from violet to yellow upon reduction of ethanol solution of colored free radical DPPH by either the process of hydrogen or electron donation. The scavenging activity was measured as the decrease in absorbance of the samples versus DPPH standard solution [19,20]. In contrast to the lower IC_50_ DPPH value of methanolic, chloroform, ethyl acetate, and aqueous extract of *V. negundo* and *V. trifolia,* the ethanolic extract of both plants have higher IC_50_ DPPH value. However, in case of hexane extract IC_50_ DPPH, the value is slightly higher as compared to the ethanolic extract of our plants [21].

Antioxidant potential needs to be supported by diverse array of assays so as to recognize the distinctive biological activities of the complex assortment of secondary metabolites [14]. Therefore, the antioxidant activity of the extracts was tested by using the other three complementary systems, β-carotene-linoleic acid, nitric oxide radical scavenging and ferrous ion chelating activity.

In the β-carotene-linoleic acid assay, linoleic acid produces hydro-peroxides as free radicals and attacks the β-carotene molecules, resulting in the reduction in the absorbance at 470 nm. β-carotene in the systems undergoes rapid discoloration in the absence of antioxidant and vice versa in its presence. The presence of different antioxidants can delay the extent of β-carotene bleaching by neutralizing the linoleate free radical and other free radicals formed in the system. Thus, the degradation rate of β-carotene-linoleate depends on the antioxidant activity of the extracts. According to Boumerfeg et al. [22], the test of linoleic acid oxidation inhibition coupled with β-carotene, appears very useful as a mimetic model of lipid peroxidation in biological membranes. β-carotene-linoleic acid assay determines the inhibition ratios of linoleic acid oxidation as further methods to confirm the anti-lipoperoxidation effects of *V. negundo* and *V. trifolia*. Lower absorbance indicates a higher level of antioxidant activity. Interestingly, in this assay, both plant extracts exhibited nitric oxide scavenging activity, which was moderately similar to each other in respect of percentage.

In the in vitro nitric oxide radical scavenging assay, nitric oxide, which responds to macromolecules, may induce inflammation. It has been stated to show a key role in numerous inflammatory processes such as carcinomas, muscle sclerosis, arthritis, and ulcerative colitis [23]. The NO scavenging effect of ethanol extracts is shown in Figure 3. It was observed that the scavenging percentage of nitric oxide was higher in the ethanol extract of *V. negundo* leaves (62.60%) and lower in *V. trifolia* leaf extract (60.87%). So, it can be interpreted that the *V. negundo* leaves have greater potential to counteract the harmful effects of NO and other reactive nitrogen species than *V. trifolia* leaves. Therefore, *V. negundo* leaves extract showed a potent scavenger of nitric oxide and thus confirmed that the plant can also be used for the treatment of anti-inflammatory diseases caused by nitric oxide formation. 

Ferrous ion chelating activity is characterized by the reduction of Fe^3+^ to Fe^2+^. The method is used to assess the effectiveness of antioxidants for their electron transfer ability. An escalation in absorbance of the reaction mixture that changes color from yellow to blue indicates an increase in the reducing capacity due to increase in the formation of the complex. Unlike the DPPH assay, the iron chelating ability of Vitex extracts is more pronounced. Figure 4 shows the reductive proficiencies of ethanol extracts of *V. negundo* and *V. trifolia* leaves compared to EDTA. It can be perceived in Figure 4 that both ethanol extracts possess certain reducing capacity, but they were less effective than EDTA. *V. negundo* possesses better reducing power, in all applied concentrations, compared to *V. trifolia* [12].

Sengul et al. [24] reported the antioxidant capacity observed, on the one hand, was not solely from the phenolic contents, but could be due to the presence of some other phytochemicals, such as ascorbic acid, tocopherol, and pigments, as well as the synergistic effects among them, which also contribute to the total antioxidant capacity. On the other hand, total phenolic contents determined according to the Folin-Ciocalteu method is not an absolute measurement of a number of phenolic materials. Different types of polyphenolic compounds have different antioxidant activities, which is dependent on their structure. The extracts possibly contain different types of phenolic compounds, which have different antioxidant capacities.

## 4. Materials and Methods

### 4.1. Plant Material

Leaves of *V. negundo* and *V. trifolia* were collected from Lucknow, Uttar Pradesh, India in September 2014. Identification and authentication were carried out by the Botany Department, and the voucher specimens (PCHNBGU/2014/56 and PCHNBGU/2014/57) were deposited in the herbarium of our Pharmaceutical Chemistry Department, H. N. B. Garhwal (A Central) University, Srinagar Garhwal, Uttarakhand, India. 

### 4.2. Chemicals and Reagents

2,2-Diphenyl-1-picrylhydrazyl (DPPH) and quercetin were purchased from Sigma Chemical Co. (St. Louis, MO, USA), while Ascorbic acid, Folin-Ciocalteu (FC) reagent, and ethanol were purchased from Thermo Fisher Scientific India Pvt. Ltd. Powai, Mumbai, India. Gallic acid, anhydrous sodium carbonate, aluminum chloride, and potassium acetate were purchased from Sisco Research Laboratory Pvt. Ltd. (Mumbai, India). All other chemicals and reagents obtained from S.D. Fine Chemicals Ltd., Mumbai, India.

### 4.3. Extraction Method

Leaves of *V. negundo* and *V. trifolia* were washed with running water and then with distilled water to remove dust and other contaminants. They were then shade dried at an average temperature of 40 °C for 84 h. Having dried, both plant materials were coarsely powdered with the help of an electric blender (Usha Pvt. Ltd., Gurgaon, India) and then passed through sieve no. 40 and stored in a closed container for further use. Different organic solvents (petroleum ether, chloroform, ethyl acetate, ethanol, and water) were used for the extraction of polar and non-polar organic compound. The powdered leaves (100 g) of *V. negundo* and *V. trifolia* were first extracted with petroleum ether using soxhlet apparatus (Borosil) for 72 h at room temperature and then successively extracted with chloroform, ethanol, and water. All extracts were concentrated and dried by using vacuum rotary evaporator (Popular Pvt. Ltd, Ambala, India) to evaporate solvents, while the concentrated extracts were kept in desiccators until further used. 

### 4.4. Qualitative Phytochemical Screening 

Phytochemical screening of active plant extracts was carried out according to the methods previously published by Mishra and Saklani [25], that is, the qualitative analysis of various phytochemicals such as alkaloids, tannins, saponins, total flavonoids and total phenols that could be responsible for antioxidant activity.

### 4.5. Determination of Total Phenolic Content (TPC)

TPC was determined using spectrophotometric method as described by Stankovic et al., 2012 [26]. In short, the reaction mixture was prepared by mixing 0.5 mL of ethanolic solution (1 mg/mL) of extract, 2.5 mL of 10% Folin-Ciocalteu’s reagent dissolved in water and 2.5 mL 7.5% NaHCO_3_. The samples were incubated at 45 °C for 15 min and absorbance was observed at 765 nm. The samples were prepared in triplicate, and the mean value of absorbance was obtained. Blank was concomitantly prepared with ethanol instead of the extract solution. The same procedure was repeated for the gallic acid, and the calibration line was constructed. The total phenolic content was expressed in terms of gallic acid equivalent (mg of GaA/g of extract).

### 4.6. Determination of Total Flavonoid Content (TFC)

TFC of the ethanolic leaf extract of both plants was measured using the aluminium chloride assay. Briefly, ethanol extract (10 mg) was dissolved in water (1 mL) in a test tube, to which 5% (*w/v*) NaNO_2_ (60 μL) was added. After 5 min, a 10% (*w*/*v*) AlCl_3_ solution (60 μL) was added. After 6 min, 1 M NaOH (400 μL) was added, and the total volume made up to 2 mL with distilled water. The solution was mixed well, and the absorbance was measured at 510 nm against a reagent blank. Concentrations were determined using a rutin standard curve. Mean total flavonoid contents (*n* = 3) were expressed as milligrams rutin equivalents (RE) per g (mg RE/g dry) [27].

### 4.7. Antioxidative Assay

#### 4.7.1. Evaluation of DPPH Scavenging Activity 

The ability of the plant extract to scavenge 2, 2-dyphenyl-2-picrylhydrazyl (DPPH) free radicals was assessed by the method described by Ćurčić et al. [28]. The stock solution of the plant extract was prepared in ethanol to achieve the concentration of 1 mg/mL. Diluted solutions (1 mL each) were mixed with DPPH (1 mL). After 30 min in darkness at room temperature (23 °C), the absorbance was recorded at 517 nm. The control samples contained all the reagents except the extract. The percentage inhibition was calculated using the following formula:

% Inhibition = (1 − A sample/A control) × 100
(1)

IC_50_ values were estimated from the % inhibition versus concentration sigmoidal curve using a non-linear regression analysis. The data were presented as mean values ± standard deviation (*n* = 3).

#### 4.7.2. Nitric Oxide Radical-Scavenging Assay 

The nitric oxide (NO) radical-scavenging activity of ethanol extracts were assayed according to Venkatachalam and Muthukrishnan, [29]. Briefly, the reaction mixture (5.0 mL) containing sodium nitroprusside (5 mM) in phosphate-buffered saline (pH 7.3), with or without the plant extract at different concentrations, was incubated at 25 °C for 3 h. The nitric oxide radical interacted with oxygen to produce the nitrite ion, which was assayed at 30-min intervals by mixing 1.0 mL incubation mixture with an equal amount of Griess reagent. The absorbance of the chromophore (purple azo dye) formed during the diazotization of nitrite ions with sulphanilamide and subsequent coupling with naphthyl ethylenediamine dihydrochloride was measured. The absorbance was measured at 546 nm by a spectrophotometer using BHT as the positive control. NO radical-scavenging activity (%) was calculated as follows:

Scavenging activity (%) = (1 − A sample/A control) × 100
(2)

#### 4.7.3. β-Carotene-Linoleic Acid Assay 

In this assay, antioxidant capacity is determined according to Katanic et al. [30] by measuring the inhibition of the volatile organic compounds, and the conjugated diene hydroperoxides arising from linoleic acid oxidation. A solution of β-carotene was prepared by dissolving β-carotene (2 mg) in chloroform (10 mL). The β-carotene-chloroform solution (2 mL) was pipetted into a round-bottomed flask, and chloroform was removed using a rotary evaporator at 40 °C for 5 min. Thereafter, 40 mg of linoleic acid, 400 mg of Tween 40 emulsifier, and 100 mL of distilled water were added to the flask with vigorous agitation to form an emulsion. The aliquots (4.8 mL) of this emulsion were added into test tubes containing different concentrations of sample solutions (0.2 mL), and the absorbance was immediately measured at 470 nm against a blank consisting of an emulsion without β-carotene. The tubes were incubated in a water bath at 50 °C. The absorbance was recorded at 20 min interval at 470 nm over a 60-min period using UV-visible spectrophotometer (Systronics India Ltd., Gujarat, India; Model No. AU-2701) at an initial time (t = 0). BHT was used as the reference compounds. 

The degradation rate (dr) of the sample was calculated according to the first order kinetics as,

dr of sample = (ln [A0/At])/t
(3)

where ln = natural log; A0 = initial absorbance at time 0; At = absorbance at 20 min of incubation; t = 120 min; and dr = degradation rate. Antioxidant activity (AA) was expressed as percent of inhibition relative to the control by using the following equation:

AA% = ([dr control − dr sample]/dr control) × 100
(4)

#### 4.7.4. Ferrous Ion Chelating Activity 

The iron-chelating abilities of the *V. negundo* and *V. trifolia* leave extracts, and standards were estimated by the method of Robu et al. [31]. In brief, four dilutions in dimethylsulphoxide (DMSO) i.e., 20 mg/mL, 10 mg/mL, 5 mg/mL, and 2.5 mg/mL were prepared from the dried extracts. Briefly, 0.05 mL of each dilution were added to a 2.7 mL TRIS buffer (pH = 7.4). Thereafter, 0.05 mL of 2 mM FeCl_2_ were added and vortexed for 15 s. At 30 s, the reaction was initiated by the addition of 5 mM of ferrozine (0.2 mL), and the mixture was shaken vigorously with the aid of cyclomixer (Remi Equipments Pvt. Ltd. Model No. CM-101, New Delhi, India) for 10 s. After 1 min beyond the addition of FeCl_2_ solution, an absorbance of the solution was measured spectrophotometrically at 562 nm. The ability of extracts to chelate ferrous ion was calculated using the following formula:

Chelating effect (%) = (1 − A sample/A control) × 100
(5)

where A is the absorbance of the control and sample (extract or standard).

The IC_50_ value (μg/mL), which is the concentration of the extract/standard that chelate 50% of the ferrous ion, was calculated through linear interpolation between values above and below 50% activity.

## 5. Conclusions

In the present study, we have made an attempt to provide the comparative antioxidant potential of phytochemicals present in the two selected *Vitex* species (*V. negundo* and *V. trifolia*). The results indicate that considerable TPC and TFC presented in the *V. negundo* and *V. trifolia* leaf extracts could be an important source of antioxidant molecules. *V. negundo* shows polyphenolic content higher than *V. trifolia*. The tested *Vitex* extracts have a strong antioxidant activity against numerous oxidative systems in vitro. It was found that *V. negundo* has a more powerful antioxidant effect than *V. trifolia*. The antioxidant capacity of polyphenols is based on their molecular structure. Therefore, our result suggests that both plant species have potent antioxidant properties. However, *V. negundo* leaf extract as compared to *V. trifolia* possesses more antioxidant potential. 

## Figures and Tables

**Table 1:** Qualitative screening of phytochemicals for selected plant extracts under different solvent systems.

Plant Part	Extract	^‡^ Carbo.	Alka.	Sapo.	Flav.	Phe.	Tan.	Terp.
***Vitex negundo* Leaves**	Pet. Ether	−	−	−	+	−	−	+
	Chloroform	−	+	−	−	+	+	−
	Ethyl Acetate	−	−	−	+	+	+	+
	Ethanol	+	−	+	+	+	+	+
	Water	+	−	+	−	−	+	−
***Vitex trifolia* Leaves**	Pet. Ether	−	−	+	−	−	−	−
	Chloroform	−	−	−	+	−	−	−
	Ethyl Acetate	−	+	−	+	+	+	+
	Ethanol	+	+	+	+	+	+	+
	Water	+	−	−	+	−	+	−

^‡^ Carbo. = Carbohydrates, Alka. = Alkaloids, Sapo. = Saponins, Flav. = Flavonoids, Phe. = Phenols, Tan. = Tannins, Terp. = Terpenoids; (+) = Presence, (−) = Absent.

**Table 2:** Secondary metabolite contents in *V. negundo* and *V. trifolia* leaves.

Parameter Analysed	*V. negundo*	*V. trifolia*
Total Phenolic Contents (mg GAE/g dry weight of extract)	89.71 ± 0.14	77.20 ± 0.22
Total Flavonoid Contents (mg QE/g dry weight of extract)	63.11 ± 0.31	57.41 ± 0.37

Each value is the average of three analyses ± standard deviation.
